# Prevalence of childhood and adolescence epilepsy in Upper Egypt (desert areas)

**DOI:** 10.1186/s41983-018-0032-0

**Published:** 2018-11-09

**Authors:** Wafaa M. Farghaly, Mohamed A. Abd Elhamed, Enas M. Hassan, Wael T. Soliman, Mohamed A. Yhia, Nermin A. Hamdy

**Affiliations:** 1 Department of Neurology, Assuit University, Assiut, Egypt; 2 Department of Neurology, Minia University, Minya, Egypt

**Keywords:** Childhood epilepsy, Epidemiology, Upper Egypt

## Abstract

**Background:**

A high prevalence of epilepsy in children is frequently found in developing countries.

**Objective:**

This study aimed to determine the prevalence and clinical pattern of childhood and adolescence epilepsy in Upper Egypt.

**Methods:**

This is a door-to-door study conducted on all inhabitants < 18 years in Al Kharga district and Al Qusier city (36,195 subjects). The study was conducted through two stages; every stage consisted of two phases (screening and diagnostic).

**Results:**

Lifetime prevalence of childhood and adolescence epilepsy (children < 18 years) in Upper Egypt was 9.7/1000, with higher prevalence among children < 12 years (10.8/1000) than adolescents (7.2/1000). The age-specific prevalence was highest in early childhood (12.01/1000) and least at adolescence (7.2/1000). More than half of the patients (59.4%) had idiopathic epilepsy. The most frequent etiology for structural/metabolic epilepsy was perinatal complications, particularly in infancy, followed by central nervous system (CNS) infections, in childhood, and post-traumatic epilepsy in adolescence. Partial seizures were more frequent in infancy, while generalized seizures were more frequent in late childhood and adolescence. Generalized tonic-clonic seizures (GTCS) were the most frequent type of seizures.

**Conclusion:**

Prevalence of childhood and adolescence epilepsy in Upper Egypt was not so much different from other developing countries. Idiopathic epilepsy was more prevalent than structural/metabolic cases. Perinatal complications, CNS infections, and head injury were the most frequent etiologies, and generalized tonic-clonic seizures were the most frequent seizure type.

**Electronic supplementary material:**

The online version of this article (10.1186/s41983-018-0032-0) contains supplementary material, which is available to authorized users.

## Introduction

A seizure represents the clinical manifestation of an abnormal excessive synchronized discharge from neurons residing primarily in the cerebral cortex. Epilepsy is a brain disorder characterized by episodes of seizures. It is not a specific disease, but rather a heterogeneous condition arising from a variety of pathological insults involving the cortex, such as tumors or genetic channelopathies [1].

Seizures and epilepsy affect infants and children more than any other age group [2]. Studies have shown that epilepsies are the most common conditions encountered in most pediatric neurology clinics in many parts of the developing world [3]. Children diagnosed with epilepsy face considerable challenges. The seizures themselves, especially when poorly controlled, may be disabling and interfere with the child’s ability to learn, whereas secondary influences, such as stigma and lack of knowledge about the condition, can negatively affect their social and psychological function [4–6].

This study aimed to determine the prevalence and clinical pattern of epilepsy among children and adolescents in Upper Egypt.

## Population and methods

### Type of the study and studied population

This is a cross-sectional door-to-door study (including every door) that was carried out on all inhabitants < 18 years (36,195 subjects) of Al Kharga district and Al Quseir city, with 50.2% males and 49.2% females included.

### The study area

The study areas are representative of two desert areas of Upper Egypt, Western Desert (Al Kharga district) and Eastern Desert (Al Quseir city). The Western Desert covers an area of 700,000 km^2^, thereby accounting around two thirds of Egypt’s total land area. The government has considered the Western Desert a frontier region and has divided it into two governorates: Matrouh to the north and New Valley to the south. The Eastern Desert is relatively mountainous. The desert environment extends all the way to the Red Sea coast (http:en.wikpedia.org/wiki/Geography-of-Egypt).

#### Instruments

An Arabic screening questionnaire (Additional file 1) was designed specifically for this project, by a group of professors of neurology, based on their knowledge and review of existing screening tools. The screening questionnaire was designed to pick up any case of major neurological disorders (epilepsy; dementia; CVS; extra pyramidal syndromes; ataxia; muscle diseases; cerebral palsy, together with Bell’s palsy; and nocturnal enuresis). The questionnaire was reviewed by 11 professors of neurology as referees, from five local Egyptian universities, and accordingly, it was reconstructed. Then, it was validated by application on 100 inpatients of the neurology department and another 100 patients from the outpatients’ neurology clinic of Assiut University Hospitals [7].

### Methods

The study was conducted on two stages: the first stage was conducted in Al Kharga district from January 1, 2006, to July 31, 2008, and the second stage was conducted in Al Quseir city from July 1, 2009, to January 31, 2012.

Each stage was conducted on two phases:

- *Phase 1*: *Screening phase*: In this stage, screening of all inhabitants was performed by three neurologists, using the constructed standardized Arabic screening questionnaire to pick up any suspected case of epilepsy [7]. Collection of demographic data was recorded by 15 female social workers who accompanied the specialists during house visits.

- *Phase 2*: *Diagnostic phase*: Any patient having a history suggestive of epilepsy was invited to attend the general hospital of Al Kharga or Al Quseir where clinical evaluation was done by other staff members with special emphasis on detailed history including age of onset, detailed semiology of the seizures from witness(s), inquiry about aura and post-ictal symptoms, seizure frequency, anti-epileptic drug(s) received, compliance to treatment, and family history of epilepsy. Full clinical evaluation was done, and inter-ictal EEG assessment and neuroimaging (CT and/or MRI) were done when needed.

If subjects were not at their homes at the first visit, they were revisited for three times later to insure their participation.

#### Case definitions used

According to the guidelines for epidemiology studies on epilepsy proposed by the Commission on Epidemiology and prognosis of the International League Against Epilepsy [8], *epilepsy* was defined as a condition characterized by two or more un-provoked seizures occurring at least 24 h apart.

Seizure types were ascertained and classified according to the classification of the ILAE, 1981 [9] and etiologic categories included idiopathic (presumably genetic), symptomatic, and cryptogenic (of unknown cause) according to [8].

### Statistical methods

The data were coded and verified prior to data entry. The Statistical Package of SPSS version 16 for Windows was used for data entry and analysis. Descriptive statistics were calculated. For qualitative data, chi-square test was used, and for quantitative data, Student’s *t* test (for two groups) was used. *Z* test was used to compare proportions and correlations. A significant *P* value was considered when *P* value was less than 0.05.

## Results

This study was carried out on 36,195 subjects, 350 out of them were diagnosed to have epilepsy with a life time prevalence of 9.7/1000. Age and sex specific prevalence of epilepsy among childhood and adolescence was demonstrated in Table 1. Table 2 shows demographic characteristic of patients with epilepsy in the studied areas. Table 3 demonstrates age of onset of epilepsy among the studied group of patients. Etiologic classification of epilepsy among the studied children and adolescents demonstrated that 31.5% of cases had symptomatic epilepsy Table 4. Table 5 illustrates the pattern and rate of different seizure types among children and adolescents with epilepsy, while Table 6 demonstrates the frequency of seizures whether severe (daily or weekly), moderate (monthly) or mild seizure frequency (one seizure per year or more) among the studied children.

**Table 1:** Age- and sex-specific prevalence of childhood and adolescence epilepsy

Item	Childhood (birth to < 12 years)			Adolescence (12 to < 18 years)	Total (birth to < 18 years)
Age group	Early (birth to < 6 years)	Late (6 to < 12 years)	Total childhood (birth to < 12 years)		
Number of population	11,987	12,577	24,564	11,631	36,195
Patients					
Number	144	122	266	84	350
Prevalence/1000	12.01	9.7	10.8	7.2	9.7
Patients					
Males					
Number	77/5958	76/6325	153/12,283	40/5877	193/18,160
Prevalence/1000	12.9	12.01	12.5	6.8	10.6
Females					
Number	67/6029	46/6252	113/12,281	44/5754	157/18,035
Prevalence/1000	11.1	7.4	9.2	7.6	8.7

**Table 2:** Demographic data of patients with epilepsy

	Infancy and early childhood (birth to < 6 years)		Late childhood (6 to < 12 years)		Adolescence (12 to < 18 years)		Total (birth to < 18 years)		*P*
	No.	%	No.	%	No.	%	No.	%	
Number	144	41.1	122	34.85	84	24.0	350	100	
Sex									0.17
Males	77	53.5	76	62.3	40	47.6	193	55.1	
Females	67	46.5	46	37.7	44	52.4	157	44.9	
Education									
Preschool age	144	100	–	–	–	–	144	41.1	0.000
Illiterate	–	–	88	72.1	24	28.6	112	32	
Primary and prep	–	–	34	27.9	28	33.3	62	17.7	
Secondary and high	–	–	–		32	38.1	32	9.0	
Residence									
Urban	107	74.3	85	69.7	69	82.1	261	74.6	0.06
Rural	37	25.7	37	30.3	15	17.9	89	25.4

**Table 3:** Age of onset of epilepsy among the studied patients

Age of onset	Number	Percentage
During infancy (birth to < 1 year)	136	38.9
During early childhood (1 to < 6 years)	144	41.1
During late childhood (6 to < 12 years)	60	17.1
During adolescence (12 to < 18 years)	10	2.9

**Table 4:** Distribution of different etiologies among studied epileptic groups

Etiology of epilepsy	Infancy and early childhood (birth to < 6 years) *N* = 144		Late childhood (6 to < 12 years) *N* = 122		Adolescents (12 to < 18 years) *N* = 84		Total *N* = 350	
	*n*	%	*n*	%	*n*	%	*N*	%
Idiopathic	76	52.8	74	60.7	58	69	208	59.4
Structural/metabolic								
Perinatal complications	29	20.1	18	14.8	10	11.9	57	16.3
CNS infection	11	7.6	10	8.2	2	2.4	23	6.6
Post-traumatic	4	2.8	2	1.6	4	4.8	10	2.9
Vascular	3	2.1	3	2.5	1	1.2	7	2
Congenital deficits	4	2.8	1	0.8	0	0	5	1.4
Metabolic	1	0.7	1	0.8	1	1.2	3	0.9
Tumors	1	0.7	2	1.6	0	0 1	3	0.9
Tuberous sclerosis	1	0.7	0	0	1	0.2	2	0.6
Unknown etiology	14	9.7	11	9	7	8.3	32	9.1

*P* < 0.0001

**Table 5:** Rate of different types of seizures among the studied epileptic groups

Types of seizures	Infancy *N* = 25		Early childhood *N* = 119		Late childhood *N* = 122		Adolescence *N* = 84		Total *N* = 350	
	*N*	%	*N*	%	*N*	%	*N*	%	*N*	%
Generalized	11	44	57	47.9	64	52.5	43	51.2	175	50
GTC	8	32	37	31.1	44	36.1	27	32.1	116	33
Tonic	2	8	16	13.4	5	4.1	10	11.9	33	9.4
Clonic	0	0	1	0.8	3	2.5	0	0	4	1.1
Absence	1	4	2	1.7	8	6.6	3	3.6	14	4
Atonic	0	0	0	0	2	1.6	2	2.4	4	1.1
Myoclonus	0	0	1	0.8	2	1.6	1	1.2	4	1.1
Partial	13	52	51	42.9	54	44.3	39	46.4	157	44.9
Simple partial	3	12	2	1.7	7	5.7	7	8.3	19	5.4
Complex partial	4	16	29	24.4	19	15.6	17	20.2	69	19.7
Partial with secondary generalization	6	24	20	16.8	28	23	15	17.9	69	19.7
Mixed seizures	1	4	11	9.2	4	3.3	2	2.3	18	5.1

*P* < 0.17

**Table 6:** Frequency of epileptic seizures among studied epileptic groups

Frequency	Infancy *n* = 25		Early childhood *n* = 119		Late childhood *n* = 122		Adolescence *n* = 84		Total *N* = 350	
	*N*	%	*N*	%	*N*	%	*N*	%	*N*	%
Daily or weekly	7	28	44	36.9	50	40.98	31	37	132	37.7
Monthly	8	32	40	33.6	41	33.6	35	41.6	124	35.4
Yearly	10	40	35	29.4	31	25.4	18	21.4	94	26.9

*P* = 0.52

## Discussion

Lifetime prevalence of epilepsy (LPE) among children and adolescents varies greatly throughout the world. Apart from differences in methodology and sample size, the variation is due to difference in the adopted definition of epilepsy, diagnostic criteria, and inclusion criteria of participants. Studies in developed countries have detected prevalence for epilepsy in children varying between 3.4/1000 in 8–11 years [10] and 5.1/1000 in 6–12 years [11]. The prevalence of epilepsy is higher in developing countries in which a prevalence of > 10 per 1000 were reported [12, 13].

In the present study, 350 patients were detected with epilepsy within the defined age group (birth to ≤ 18 years), of whom 193 were males (55.1%) and 157 females (44.9%). Age-specific lifetime prevalence of epilepsy among children and adolescents (< 18 years of age) was 9.7/1000. It was higher (12.01/1000) during early childhood (birth to < 6 years of age) than among late childhood (9.7/1000) and adolescence (7.2/1000) (Table 1). A lower rate was reported in Minia city by Hamdy [14], who reported that the lifetime prevalence of epilepsy among primary school children was 7.2/1000. Similarly, El-Motayam [15] found a prevalence of 7–10/1000 among school children below the age of 15 years, while higher rates reported by Shawki and colleagues [16] who found that the prevalence of epilepsy among school children (≤ 12 years of age) was 12.9/1000 in Upper Egypt, Asyut Governorate. Egyptian studies are much lower than that reported among 5-year-old Brazilian children (45.2/1000) [17] and among 12-year-old school children in Tahran (32.4/1000) [18]. This high prevalence reported in the Brazilian and Tahran studies could be explained by difference in the adopted definition of epilepsy where both studies recorded lifetime prevalence of seizure (LPS) not for epilepsy, and they did not necessitate seizure recurrence. Accordingly, our estimated lifetime prevalence of epilepsy in the current study is lower than that recorded in one of the most developed countries, USA, where a prevalence of epilepsy/seizure disorder among children (birth to 17 years old) was estimated to be 10.2/1000 [19].

Regarding sex, it was found that the prevalence of epilepsy was higher among males (10.6/1000) than females (8.7/1000) (Table 1). This observation was true along the whole childhood period (12.5/1000 versus 9.2/1000), but the reverse was apparent during adolescence where the prevalence was higher among females (7.6/1000) than males (6.8/1000). Most studies of sex distribution among children with epilepsy describe a preponderance of boys [10, 18, 20]. This higher prevalence of epilepsy among boys during infancy and childhood period might reflect deeply seated male sex preference, at least in our country, and consequently, their parents seek advice for this precious baby or child. However, during adolescence, and with the approaching of the girl’s age of marriage, her family starts to seek advice for her recurrent seizure problem.

In the present study, 41% (NO = 144/350) of the patients’ sample were at the preschool age (< 6 years) (Table 2). Of children and adolescents at school age (NO = 206/350), more than half (*n* = 112/206; 54.4%) were illiterate. This could reflect the severe impact of the disease and/or the underlying etiology, together with the effect of the concurrently administered anti-epileptic drugs, on the cognitive function of the affected children and adolescents.

It has been estimated that in developing countries, 60–75% are living in rural areas, while in developed countries, 25% of the population live in rural areas. Accordingly, Kandil and colleagues [21] found that 78% of children and adolescents with epilepsy in Asyut, Egypt, were rural residents. Although Egypt is considered one of the developing countries, the reverse is applied to the two studied areas: Al Khargah and Al Qusier city, both of which are considered new urbanized areas, and this could explain the higher rate of epilepsy in urban (74.6%) than rural areas (25.4%) in the present study.

In the present research, most of the studied cases (80%) reported onset of their seizures during infancy or early childhood (< 6 years) (Table 3). The high incidence of seizure onset during infancy and early childhood (80%) than that in late childhood (17%) and adolescence (2.9%) might be attributed to a decreased seizure threshold of the immature brain, which may be in part due to paradoxical excitation of GABA during early brain development, while GABA causes hypopolarization and inhibition of neurons in adulthood. Moreover [22], the higher incidence of brain insults at that age including perinatal complications and CNS infections, together with genetic defects, all initiate epileptogenesis in the immature S brain. Similarly, in a comparative prospective study carried on children of Saudi Arabia by Al-Sulaiman and Ismail [23], the recorded age of onset was within the first year of life in 48.7% of the patients. On the contrary, Kramer [24] in a consecutive study from Israel found that 18% of the seizures began in infancy, 64% in childhood period, and 18% in adolescents.

Epilepsy was found to be idiopathic in 59.4% of epileptic children (Table 4). This is consistent with many studies, where Shawki and colleagues [16] in Asyut, Egypt, found no etiology for epilepsy in 58.2% of patients, and in Lithuania, no etiology was identified in 60.3% of patients [25]. This high rate of presumably genetic epilepsy might be attributed to the higher rate of consanguineous marriage in these areas.

In this study, 31.6% had structural/metabolic causes of epilepsy, and the impact of perinatal complications stands as the commonest cause among all age groups of the studied patients (Table 4). This is nearly similar to that reported by Bielmann and colleagues [26] who found symptomatic epilepsy among children in Estonia to be 40.7% of patients, out of which perinatal events were the most frequent etiology. Moreover, Kwong and colleagues [27] reported symptomatic epilepsy in 61% of a cohort of 309 Chinese children, and perinatal factors were the most frequently encountered cause of epilepsy. This high rate of perinatal complications could be attributed to the low socioeconomic state and lack of antenatal and perinatal care with consequently high frequency of birth injuries and hypoxic ischemic encephalopathies.

Regarding seizure type, partial seizures were more frequent in infancy (52%), while generalized seizures were more frequent in childhood and adolescence without statistically significant difference (Table 5). This is in agreement with Endziniene and colleagues [25] who demonstrated that 50% of their pediatric patients with epilepsy were localization-related, while only 29.9% were generalized epilepsies, 15.9% were undetermined whether partial or generalized, and 4.2% were unclassifiable. Moreover, in a study in Southern Stockholm, Sweden, Braathen and Theorell [28] recorded partial seizures in 52% of the children with epilepsy. On the other hand, Al Rajeh and colleagues [29] in Saudi Arabia demonstrated that generalized epilepsies were the commonest type of epilepsy between ages of 1 and 5 years (74%).

Generalized tonic-clonic (GTC) seizures were the most frequent type of seizures among children (33%), while absence seizures were recorded in 4% only. Similarly, Shawki and colleagues [16] demonstrated that generalized tonic-clonic seizures were the most frequently encountered type of seizures (49.5%), while absence (1.0%) was the least frequent type. Furthermore, Granieri and colleagues [30] reported tonic, clonic, and tonic-clonic seizures in 74.9% and childhood absence in 6.6% of epileptics. In partial agreement, Kandil and colleagues [21] identified four subtypes of generalized epilepsy, among which the generalized tonic-clonic seizures were the most frequent (72.1%), followed by absence (14.9%). Muhammad and colleagues [31] also found that the most common type of epileptic seizures was generalized tonic-clonic seizures. This low rate of absence epilepsy could be attributed to the fact that absence seizures could pass unnoticed and may not be easily recognized.

In the current study, 37.7% of epileptic children had daily or weekly seizures. This frequency was the most commonly encountered one among early and late childhood. It tends to regress slightly among adolescence where monthly seizures became the most prevailing one. This is in agreement with Hamdy [14] who found that the highest frequency of seizures was once or more per week (47.3%), followed by seizures occurring once or more per month (24.3%) and once or more per day (18.9%). Salih and colleagues [32] reported that age was not a driving factor to influence the seizure frequency in the studied patients. Moreover, a study conducted in the USA demonstrated no significant correlation between the changes in the seizure frequency and age [33].

As we have no accurate data registry in our country, door-to-door community study is the best method to study the prevalence of childhood and adolescent epilepsy. Also, the presence of screening questionnaire may help in performing this type of study. On the other hand, there are some limitations in the present study, i.e., the studied areas are far away from our university, some security issues, and poor health services in the studied areas.

## Conclusion

Prevalence of epilepsy in this region (9.7/1000) is not so much different from other developing countries. The most common (59.4%) underlying cause of epilepsy was the idiopathic type, while the most frequent etiology for structural/metabolic epilepsy was perinatal complications along all studied age groups. Focal seizures were frequent in infancy while generalized seizures were more frequent in childhood and adolescence. The generalized tonic-clonic seizures were the most common seizure type among children and adolescents. This knowledge will facilitate in the diagnosis, early educational intervention, and multidisciplinary therapeutic and rehabilitation approaches.

## Additional file

Additional file 1: An Arabic screening questionnaire. (DOCX 17 kb)
