# Supplementary material for: Prevalence of childhood and adolescence epilepsy in Upper Egypt (desert areas)
Source: Egypt J Neurol Psychiatr Neurosurg. 2018 Nov 9;54(1):34. doi: 10.1186/s41983-018-0032-0 (PMC6245138; doi:10.1186/s41983-018-0032-0)
Supplement: Supplementary file 1 — An Arabic screening questionnaire. (DOCX 17 kb) [file 41983_2018_32_MOESM1_ESM.docx]

**الخامس**

**الرابع**

**الثالث**

**الأول**

**الثانى**

1. هل عانى الطفل أو الطفلة من تأخر فى الوظائف العقلية مثل الابتسام أو التعرف على وجه الأم أو تعلم الكلام (للأطفال)؟

2. هل عانى الطفل أو الطفلة من تأخر فى النمو الحركى مثل الجلوس أو الحبو أو الوقوف أو المشى؟

3. هل حدث أن فقدت الوعى أو لم تشعر بمن حولك؟

4. هل عانيت من حدوث تشنجات عصبية مصاحبة لفقد فى درجة الوعى أو لا؟

5. هل توجد أى حركات لا إرادية (رعشة) فى أى جزء من الجسم؟

6. هل تعانى من اضطراب فى النوم أو من حدوث حركات لا إرادية أو حدوث تبول لا إرادى أثناء النوم؟

7. هل تعانى من نقص مفاجئ أو حدوث فقد مفاجئ للبصر فى إحدى العينين أو كلاهما أو حدوث ازدواج فى الرؤية؟ أو ارتخاء فى جفن العين أو عدم القدرة على غلق إحدى العينين أو كلاهما؟

8. هلى تعانى من نوبات تشم فيها رائحة غريبة؟

9. هل تعانى من ضعف فى السمع أو تعانى من نوبات إحساس بالدوار؟

10. هل تعانى من صعوبة فى مضغ أو بلع الطعام ؟ فى حالة نعم – هل يوجد ارتجاع للماء من الأنف أو شنجة (شرقة)؟

11. هل يعانى المريض من صعوبة فى النطق أو من مشكلات فى الكلام؟ أو فى فهم ما يقال حوله (informant)

12. هل تعانى من ضعف أو ثقل فى أى من الأطراف الأربعة؟

13. هل تعانى من مشكلات فى استخدام اليدين فى أداء بعض المهام الدقيقة مثل الكتابة على الكمبيوتر أو ما شابة ذلك أو استخدام أبره الخياطة ، مسك مشط الشعر ، استخدام المقص أو السكين أو الملاعق؟

14. هل تعانى من وجود صعوبة فى المشى أو تكرار الوقوع أثناء المشى؟ أو ترنح أثناء المشى؟

15. هل تعانى من بطئ فى الحركة أو شعور بالتكتيف أثناء الحركة؟

16. هل تعانى من شعور بالتنميل أو التخدير أو ألم فى الوجه أو أى من الأطراف الأربعة أو فى أى جزء من أجزاء جسمك؟

**الثانى**

**الأول**

**الخامس**

**الرابع**

**الثالث**

17. هل تشعر بالأرض تحت قدميك أثناء المشى أو باضطراب المشى فى الضوء الخافت اضطراب الاتزان عند غلق العينين؟

18. هل تعانى من وجود مشاكل فى التحكم فى البول أو البراز؟

19. هل تعانى من صعوبة فى ارتداء الملابس أو تمشيط الشعر أو القيام من وضع الجلوس (على الأرض أو على المرحاض) إلى وضع الوقوف (مع عدم الإحساس بآلام فى الركبتين)؟
